# Continuous sample drop flow microextraction: a versatile and green microextraction tool in analytical chemistry

**DOI:** 10.1039/d6ra02146h

**Published:** 2026-06-02

**Authors:** Anwar Rasheed Yaqoub, Sameera Sh. Mohammed Ameen, Anwar H. Abdullah, Khalid M. Omer

**Affiliations:** a Department of Pharmacy, College of Pharmacy, Nawroz University 42001 Duhok Kurdistan Region Iraq; b Department of Chemistry, College of Science, University of Zakho 42002 Zakho Kurdistan Region Iraq; c Department of Chemistry, College of Science, Duhok University 42001 Duhok Kurdistan Region Iraq; d Department of Chemistry, College of Science, University of Sulaimani 46002 Sulaymaniyah Kurdistan Region Iraq khalid.omer@univsul.edu.iq

## Abstract

Continuous sample drop-flow microextraction (CSDF-ME) has become a powerful and versatile liquid-phase microextraction methodology that overcomes several of the analytical and environmental shortcomings of traditional sample preparation procedures. In CSDF-ME, the aqueous sample is continuously added to a small, stagnant volume of extraction solvent, resulting in large renewed interfacial areas that favor efficient mass transfer, high enrichment factors, and good reproducibility, using only microliter volumes of solvent. Despite its growing importance and increasing range of applications, an independent and comprehensive review dedicated exclusively to CSDF-ME is still lacking; therefore, a critical and focused overview of this technique is highly necessary. This review provides a comprehensive overview of CSDF-ME, covering its working principles, method development and optimization strategies, analytical performance and validation, as well as its applications in environmental, food, biological, and pharmaceutical analyses. Special focus is given to recent advances in green extraction methods, including the use of fatty acids and deep eutectic solvents. The existing constraints, such as automation and solid-sample processing, are addressed, and the prospects for further evolution and broader use of CSDF-ME are outlined. In general, CSDF-ME has proven to be an effective and environmentally friendly sample preparation method in modern analytical chemistry.

## Abstract 2

Continuous sample drop-flow microextraction (CSDF-ME) has become a powerful and versatile liquid-phase microextraction methodology that overcomes several of the analytical and environmental shortcomings of traditional sample preparation procedures.

## Graphical abstract

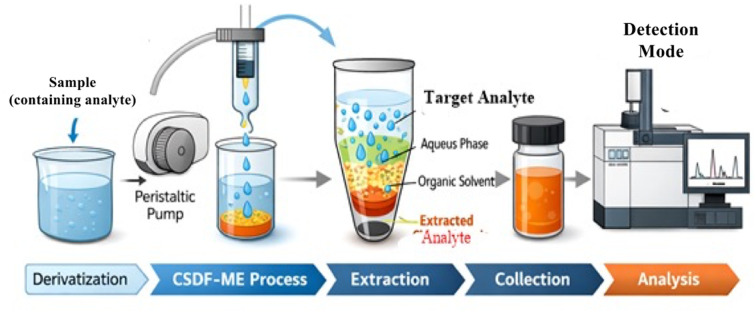

## 1. Introduction

One of the most time-consuming and critical processes in analytical chemistry is sample preparation, especially for the determination of trace-level analytes in complex samples such as environmental waters, food products, biological fluids, and pharmaceutical samples. Traditional methods such as liquid–liquid extraction (LLE) and solid-phase extraction (SPE) are commonly used, but they tend to consume large amounts of solvents, require numerous processing steps, need considerable extraction time, and often provide limited enrichment, which is not in line with the principles of modern and green analytical chemistry.^1–3^

Compared with conventional liquid–liquid extraction (LLE) and solid-phase extraction (SPE), CSDF-ME offers clear advantages in terms of solvent consumption, operational simplicity, and sample throughput.^4–6^ LLE often requires relatively large volumes of organic solvents and multiple extraction or phase-separation steps, while SPE commonly involves cartridge conditioning, sample loading, washing, and elution, which can increase solvent use and analysis time.^4,6^ In contrast, CSDF-ME uses only microliter volumes of extraction solvent and relies on continuous droplet–solvent contact to enhance mass transfer and preconcentration.^7–9^ This makes CSDF-ME more compatible with the principles of green analytical chemistry and more attractive for routine trace-level analysis.^5,7,8^ Recent reviews on green sample preparation and liquid-phase microextraction have also emphasized the increasing importance of solvent-minimized techniques, eco-friendly extraction phases, automation, and deep eutectic solvent-based systems in modern analytical chemistry.^4,5,10^ To overcome these disadvantages, liquid-phase microextraction (LPME) methods have been widely introduced as miniaturized and environmentally friendly alternatives. Techniques such as single-drop microextraction (SDME), hollow-fiber liquid-phase microextraction (HF-LPME), dispersive liquid–liquid microextraction (DLLME), and continuous-flow microextraction (CFME) greatly minimize the amount of solvent used for extraction while providing high preconcentration efficiency.^11–16^ However, these methods can still be limited by factors such as droplet instability in SDME, the use of disperser solvents and centrifugation in DLLME, comparatively long extraction times in HF-LPME, and issues related to reproducibility and automation.^12–14,17^

To overcome some of these limitations, a new approach was introduced under the LPME family, known as continuous sample drop-flow microextraction (CSDF-ME).^7,18^ In CSDF-ME, the aqueous sample is continuously introduced into a small, stationary volume of extraction solvent in the form of fine droplets, generating a large and continuously renewed interfacial area. This configuration enhances mass-transfer efficiency, enables high enrichment factors, and ensures excellent solvent stability. The use of controlled flow, typically provided by a peristaltic pump, also allows semi-automation and improved repeatability compared with conventional droplet-based techniques.^7,8^

Since its discovery, CSDF-ME has gained increasing attention because of its simplicity, low solvent consumption, enrichment capability, and compatibility with various analytical detection methods, such as gas chromatography (GC), high-performance liquid chromatography (HPLC), atomic absorption spectrometry (AAS), and mass spectrometry (MS), among others.^7–9,19,20^ The technique has been effective for the determination of organic pollutants, pesticides, metal ions, surfactants, and pharmaceutical compounds in a wide variety of matrices, including water, food, biological fluids, and industrial products, and, therefore, is an excellent general-purpose sample preparation methodology.^8,9,19–22^

More recently, increasing attention has been directed toward enhancing the environmental sustainability of CSDF-ME by replacing traditional halogenated solvents with more environmentally friendly alternatives. The incorporation of fatty acids and deep eutectic solvents as extraction media has further improved the alignment of CSDF-ME with the principles of green analytical chemistry, while maintaining, or even improving, its analytical performance without compromising efficiency.^23–25^

The extensive growth of applications and methodological advances in CSDF-ME highlights the need for a critical assessment of this technique. This review systematically discusses the basic principles of CSDF-ME, recent developments in method development, and optimization strategies. It also critically evaluates analytical performance properties, such as sensitivity, precision, accuracy, and robustness, and reviews a broad spectrum of reported applications in environmental, food, pharmaceutical, and biological analyses. The existing limitations and practical issues related to CSDF-ME are also discussed, along with emerging trends and future research directions. Altogether, this review emphasizes that CSDF-ME can be used as a flexible, effective, and eco-friendly sample preparation method, which has become increasingly important in modern analytical chemistry.

## 2. Principles and mechanism of CSDF-ME

**Fig. 1:**
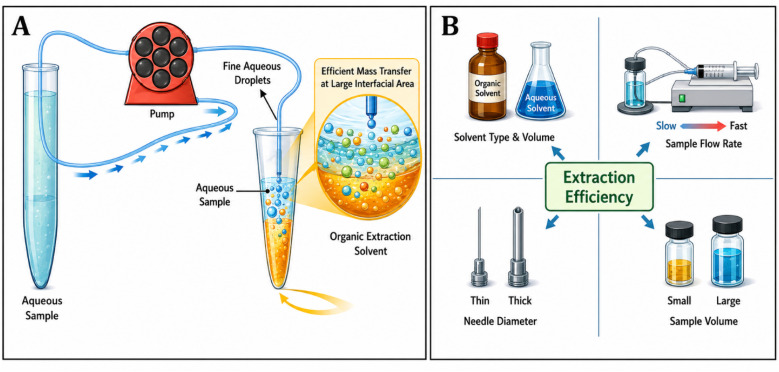
(A) Schematic diagram showing the components of CSDF-ME. (B) Key parameters influencing extraction efficiency.

CSDF-ME is based on continuous contact between an aqueous sample and a small volume of immiscible extraction solvent by introducing the sample into the extraction phase as a stream of fine droplets (Fig. 1A).^7^ In a typical setup, a microliter-scale volume of extraction solvent is placed in a conical-bottom vial. The aqueous sample is then pumped through a needle positioned within (or just above) the extraction solvent. As aqueous droplets form and pass through the organic phase, analytes partition into the extraction solvent across a large interfacial area, and the continuous renewal of droplets sustains efficient mass transfer.

Compared with conventional CFME designs, CSDF-ME maintains the extraction solvent as a stationary, discrete phase, which simplifies solvent handling and can improve operational stability. Relative to classical direct-immersion SDME formats where a microdrop may be exposed at the needle tip, CSDF-ME avoids hanging-drop dislodgement as a primary failure mode and can therefore offer improved repeatability under controlled flow conditions.^7,11,26^ At the same time, it should be recognized that SDME and DLLME include multiple modern variants, and comparisons should be made with reference to the specific configuration rather than by assuming a single classical workflow.^8^

### 2.1. Key parameters governing extraction efficiency

The extraction performance of CSDF-ME is primarily influenced by parameters that control (i) partitioning thermodynamics and (ii) mass-transfer kinetics. The most influential experimental factors typically include (Fig. 1B):^8^

The nature of the extraction solvent, including polarity, viscosity, density, analyte affinity, and instrumental compatibility. Extraction solvent volume, which affects phase ratio and attainable enrichment.

• Sample flow rate, which influences droplet formation frequency, residence time, and convective transport.

• Needle inner diameter/geometry, which affects droplet size distribution and interfacial area.

• Total sample volume processed, which determines the total analyte mass delivered to the interface.

• Matrix conditions, including pH, ionic strength, and the presence of surfactants/particulates that may affect droplet formation and partitioning.

In practice, these parameters are interdependent: for example, increasing the flow rate may increase droplet generation and convective mass transfer but can also alter droplet size and reduce individual droplet residence time, potentially changing the net extraction behavior. Optimization therefore commonly targets a balance between stable droplet formation, sufficient droplet–extractant contact, and minimized solvent loss.

### 2.2. Post-extraction handling: how the extractant is isolated and collected

**Scheme 1:**
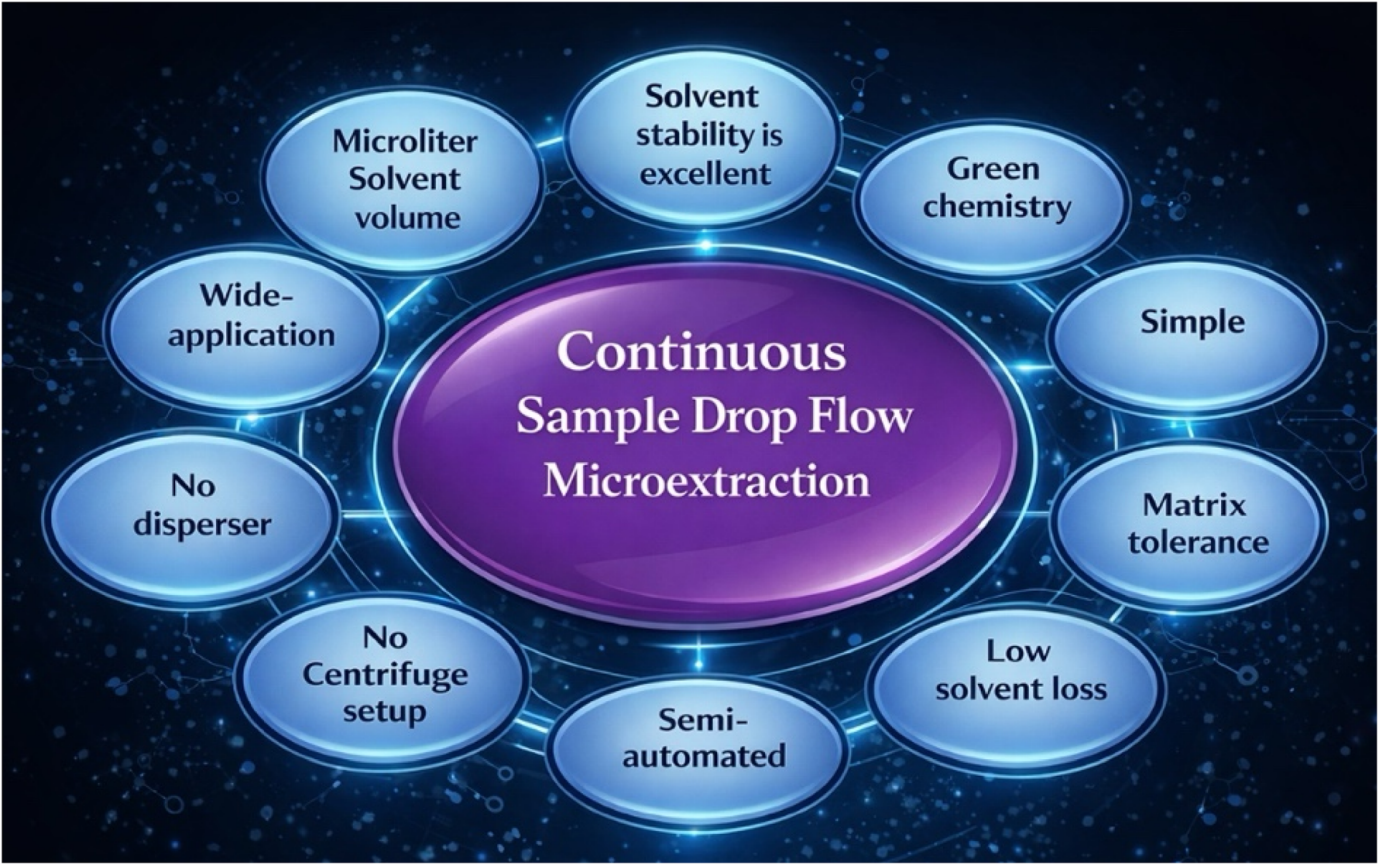
Scheme showing the common advantages of CSDF-ME.

A practical advantage of CSDF-ME is that the extraction solvent remains as a single, recoverable microvolume throughout the procedure rather than forming a persistent emulsion. After extraction, the organic phase is typically collected directly using a microsyringe or a micropipette and then transferred for instrumental introduction, such as gas chromatography (GC) injection, an HPLC vial, or compatible interfaces (Scheme 1).^7,8^

The specific collection approach depends mainly on solvent density and vial geometry. High-density extractants, which are denser than water, tend to remain at the bottom of a conical vial, facilitating microsyringe aspiration. Low-density extractants, which are less dense than water, may float; collection is still feasible using appropriate vial designs and careful aspiration at the phase boundary. In some instrumental couplings, the extraction phase may be recovered with minimal handling by positioning the vial for automated withdrawal/injection, supporting improved repeatability and reduced solvent loss.

This direct phase collection is distinct from many dispersion-based workflows where a separate demulsification or phase separation step is often required.

## 3. Comparison with other LPME techniques

CSDF-ME shares the general goals of LPME, namely low solvent consumption and effective enrichment, but differs in how interfacial area is generated and how the extractant phase is handled. Importantly, major LPME families such as SDME, DLLME, and HF-LPME have diversified into multiple configurations over time; therefore, comparisons should be framed as configuration-dependent rather than based exclusively on early “classical” workflows.^27–29^

At a broad level, technique-level comparisons are most informative when based on workflow and operational characteristics that are relatively independent of detector choice, such as: the number of handling steps and ease of phase recovery; solvent consumption and solvent integrity/stability during extraction; robustness to matrix complexity (*e.g.*, particulates, and surfactants); and compatibility with semi-automation and routine use.^30,31^

In contrast, absolute analytical figures of merit such as the limit of detection (LOD) and linear range are strongly dependent on the downstream detector such as GC-MS, HPLC-UV or AAS, analyte chemistry, and matrix effects. Consequently, these parameters are best discussed in application-specific case studies under harmonized detection conditions rather than used for generalized cross-technique ranking (Table 1).^32,33^

**Table 1:** Workflow-oriented comparison of CSDF-ME with major LPME families, including classical formats and common modern variants^a^

Technique family	Generation of interfacial area	Phase handling after extraction (separation & recovery)	Automation potential	Typical practical considerations
CSDF-ME	Successive aqueous droplets traverse a stationary extractant microvolume (renewed interface)	No disperser solvent; no centrifugation. Extractant collected directly (microsyringe/micropipette)	Moderate (pump-assisted; semi-automated)	Droplet regime control; clogging in dirty matrices; verify recovered extractant volume for reproducible enrichment
SDME (classical + stabilized variants)	Single microdrop exposed to sample (direct immersion) or headspace	No disperser solvent; no centrifugation. Drop withdrawn into syringe; stabilized/holder-based handling in some variants	Low–moderate (configuration-dependent)	Classical direct-immersion formats can suffer drop instability and operator dependence; stabilized variants reduce these issues
DLLME (classical + disperser-less/centrifuge-less variants)	Extractant dispersed into sample to form microdroplets (large area rapidly)	Classical: disperser solvent and centrifugation often used; modern variants may be disperser-less and/or centrifuge-less. Microphase collected after coalescence (variant-specific)	Moderate (some variants more automation-friendly)	Workflow and robustness depend on dispersion + separation strategy; emulsion/coalescence control can be critical
HF-LPME	Membrane-supported interface; diffusion into acceptor phase	No disperser solvent; no centrifugation. Acceptor phase withdrawn from fiber lumen	Low–moderate	Strong cleanup/selectivity; may require longer equilibration; fiber handling and fouling considerations
CFME/continuous-flow formats	Continuous contact between flowing sample and extractant interface	No disperser solvent; no centrifugation (device-dependent). Extractant recovery depends on device design	Moderate	Device complexity; reproducibility depends on controlled fluidics and interface stability

a The table intentionally emphasizes workflow and handling. Absolute LOD and linear range values are detector-, analyte-, and matrix-dependent and are therefore not included as general comparison metrics.

### 3.1. CSDF-ME relative to SDME

In classical SDME, a microdrop serves as the extraction phase, commonly positioned at the needle tip for direct immersion or exposed to the headspace. While SDME is solvent-minimizing and conceptually simple, classical hanging-drop formats can be sensitive to mechanical instability, agitation, and operator handling.^27^ CSDF-ME, by maintaining the extractant as a stationary microvolume and delivering the sample as droplets, can reduce the likelihood of drop dislodgement and can improve repeatability when droplet formation is well controlled. Nevertheless, stabilized SDME variants exist and may alleviate some classical limitations, so method selection should consider the specific SDME configuration and intended throughput.^7,8^

### 3.2. CSDF-ME relative to DLLME (including centrifuge-less/disperser-less variants)

DLLME is characterized by dispersing the extraction solvent into the sample to create a large interfacial area rapidly. In its original form, dispersion was frequently achieved using a disperser solvent and phase collection was often facilitated by centrifugation. However, modern DLLME procedures may avoid one or both of these elements through alternative dispersion strategies such as vortexing, ultrasound, or air assistance, and alternative phase-separation/collection strategies.^34^

In the context of modern DLLME variants, “centrifuge-less” typically refers to replacing centrifugation with other phase-separation/collection mechanisms such as salting-out-driven demulsification, flotation-based collection, or other physical/chemical demulsification strategies. Because these variants can differ markedly in complexity and robustness, DLLME should not be described universally as requiring disperser solvent and centrifugation; instead, the relevant DLLME variant should be specified during comparison.^29^

CSDF-ME differs fundamentally in that it preserves the extraction solvent as a discrete microphase and typically enables phase recovery by direct microsyringe collection, without the need for an emulsion-breaking step. This can simplify the workflow and support semi-automation in routine settings.^7^

### 3.3. CSDF-ME relative to HF-LPME and CFME

HF-LPME provides a membrane barrier that can offer excellent selectivity and cleanup, particularly for complex matrices, but may require longer extraction times due to membrane-limited mass transfer.^28^ CSDF-ME can provide faster extraction in many implementations by eliminating the membrane diffusion barrier and by continually renewing the droplet–extractant interface.^7,8,19^ CFME shares the continuous-contact concept, but CSDF-ME's discrete stationary extraction phase can simplify solvent handling and recovery while maintaining efficient mass transfer.

### 3.4. Summary of comparative positioning

Overall, CSDF-ME offers a practical combination of (i) microliter-scale solvent consumption, (ii) efficient mass transfer *via* continuously renewed droplet interfaces, (iii) stable extractant handling, and (iv) compatibility with pump-assisted semi-automation.^7,19^ No single LPME technique is universally optimal; selection depends on matrix complexity, analyte properties, required throughput, and the intended analytical platform. Within this landscape, CSDF-ME is especially attractive for routine and higher throughput workflows where robustness, simplified phase recovery, and reduced solvent use are priorities.

## 4. Method development and optimization

**Scheme 2:**
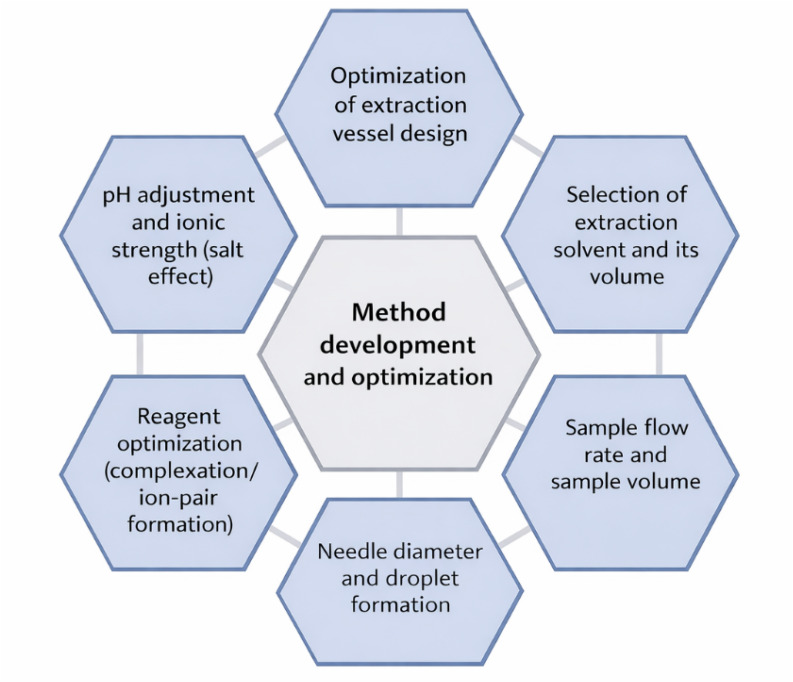
Schematic illustration of the method development and optimization process of CSDF-ME.

CSDF-ME enhances traditional droplet-based LPME by actively driving the aqueous sample through a small, stable extraction phase using a peristaltic pump. In this approach as shown in Scheme 2, the extraction solvent is held in a conical vessel, while the sample is delivered as a series of continuous droplets that pass through the solvent phase. This repeated droplet–solvent interaction promotes effective mass transfer and extraction while preserving a constant microvolume of the extractant. As a result, the configuration improves method repeatability, since the extraction solvent remains stable and the process is partially automated through controlled pumping.^7^ A peristaltic pump is central for ensuring a steady droplet formation and reproducible contact time; for example, stable flow control within the range of 0.4–8.0 mL min^−1^ range depending on study has been reported in multiple CSDF-ME platforms.^8,35^

### 4.1. Optimization of the extraction vessel design

**Fig. 2:**
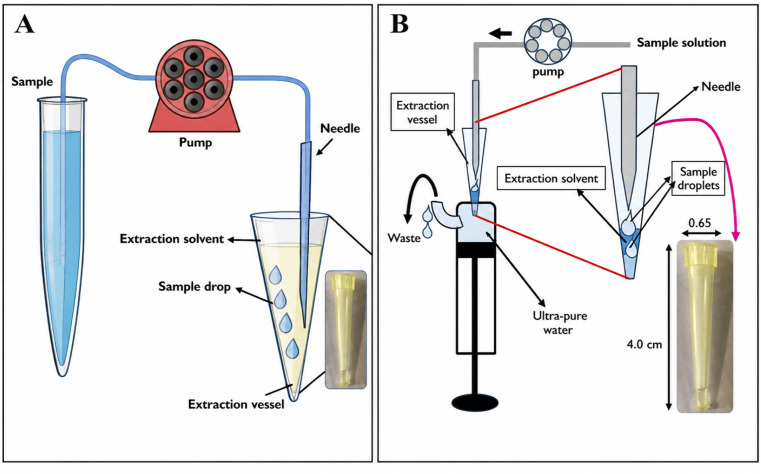
(A) CSDF-ME procedure with a modified extraction vessel. This figure has been adapted/reproduced from ref. 9 with permission from Elsevier, copyright 2020. This vessel redesign allowed the use of low-density solvents, *i.e.*, solvents less dense than water, by placing the conical open-end vial in a water-filled container so that solvents such as hexane could be retained at the top while sample droplets passed through the organic layer. (B) Scheme of the proposed CSDF-ME procedure. This figure has been adapted/reproduced from ref. 36 with permission from Elsevier, copyright 2021.

The geometry of extraction vessels has a strong effect on the droplet rise path, droplet residence time and consequently enrichment (Fig. 2). A modified CSDF-ME design with a narrow-necked conical vessel was demonstrated to have a significant impact on droplet formation and extraction performance. Internal diameter (ID) and conical-section height were compared in a variety of vessel designs in that study, and the vessel with a longer conical section and smaller ID was found to give a higher EF because it prolonged the droplet–solvent contact time during the ascent of the droplet.^9^

### 4.2. Selection of extraction solvent and solvent volume

The selection of the solvent is usually informed by (i) immiscibility with water, (ii) adequate solubility of the analyte/partitioning, (iii) stability under continuous droplet flow, and (iv) compatibility with the final detection system. In the case of organophosphorus pesticides (OPPs) in juice/water samples, chloroform performed better than the other choices, including chlorobenzene and dichloromethane, with respect to repeatability, sharp peaks, high EF, and low RSD In the case of ion-pair CSDF-ME for surfactant–dye extraction, a wide range of dense solvents was screened, including CCl_4_, chloroform, DCM, chlorobenzene, bromobenzene, and CS_2_, with chloroform giving the highest analytical response.^35^ One of the trade-off parameters is the volume of extraction solvent: a small volume can reduce the absolute extracted mass, whereas a large volume can increase the final extract volume at the expense of enrichment. The volume of chloroform (80–110 µL) was adjusted in the surfactant application to balance EF, sensitivity, and solvent consumption.^35^ For the modified OPP method, microvolumes of chloroform were used for example, 12 µL, demonstrating that very low extractant volumes can still support strong enrichment when droplet contact is efficient.^9^

A key evolution in CSDF-ME method development is the replacement of conventional halogenated solvents with greener alternatives. One approach used octanoic acid a green solvent as the extraction phase, and optimization explicitly included pH, solvent volume, sample volume, salt, acid addition, and flow rate to reach best performance for antibiotics in urine with using HPLC detection.^22^ Another major direction is introducing deep eutectic solvents (DESs) to further align CSDF-ME with green chemistry goals. In that work, multiple DES compositions were synthesized and screened, subsequently practical CSDF-ME variables, including pH, sample volume, flow rate, and HPLC conditions (mobile phase type/composition) were optimized to maximize antibiotic extraction from wastewater and urine.^21^

Additionally, centrifuge-less and switchable-solvent CSDF-ME variants have been reported such as CL-CSDF-ME where experimental factors were screened and optimized using chemometrics software, strengthening robustness and method transferability.^37^

### 4.3. Needle diameter and droplet formation control

The effect of needle dimensions on interfacial area and mass transfer is directly related to their influence on droplet size and frequency. In the CSDF-ME of surfactants, the optimum response was attained using a needle with an external diameter of 0.3 mm, which implies that droplet formation stability and surface area were maximized with this geometry.^9^

### 4.4. Sample flow rate and sample volume

Flow rate defines the contact time between droplets and the organic phase. Optimization studies consistently show that increasing the flow rate shortens droplet–solvent interaction time and reduces EF after a certain point. For OPPs, flow rate evaluation within the range of 0.5–0.9 mL min^−1^ showed decreased extraction at higher flow rates, and 0.5 mL min^−1^ was selected as the optimal value because EF dropped as a result of reduced contact time at higher flow rates.^9^

In the surfactant technique, the maximum absorbance was obtained at 0.5 mL min^−1^; nevertheless, a higher flow rate, 1.0 mL min^−1^, was selected as a practical compromise to minimize analysis time in the remaining procedures.^35^

Sample volume influences preconcentration because a larger processed volume contains more analyte mass available to be transferred into the microvolume solvent, but it increases extraction time at a fixed flow rate. For OPPs, EF increased when the sample volume rose from 6 to 10 mL, but extraction time increased correspondingly; therefore, 8 mL was selected as a practical optimum.^9^

### 4.5. pH adjustment and ionic strength (salt effect)

pH regulates the ionization of analytes and/or complexation, and therefore, has a significant impact on extraction. Extraction recovery in metal-ion CSDF-ME with APDC chelation was constant and maximized in the pH range of 3–8, with pH 3 being used to prevent complex instability at very acidic pH and precipitation at the very alkaline pH.^38^

Likewise, Pb^2+^ extraction was found to exhibit nearly constant recovery over a narrow acidic range, 2.5–3.5, and pH 3.0 was chosen for subsequent experiments.^19^

Conversely, in the case of antibiotic drug determination, the acid–base characteristics and p*K*_a_ values of the target compounds govern pH optimization rather than chelation chemistry. The antibiotics levofloxacin, metronidazole, and tinidazole have ionizable functional groups, and their extraction efficiency is sensitive to the ionization state. CSDF-ME studies have shown that alkaline conditions tend to be necessary to suppress protonation, increase hydrophobicity, and improve partitioning into the extraction solvent. For example, levofloxacin was found to be extracted most efficiently at very alkaline pH, approximately 11, whereas metronidazole and tinidazole were most effective at slightly alkaline to neutral pH. Therefore, in contrast to CSDF-ME methods targeting metal ions, antibiotic determination normally requires neutral to alkaline pH conditions to favor the molecular, non-ionized form and increase the efficiency of the extraction process.^21,22^

This comparison shows the flexibility of CSDF-ME because the method can be easily extended to other classes of analytes *via* proper pH control. Whereas metal-complexation-based CSDF-ME requires acidic conditions, alkaline or near-neutral conditions are more appropriate for antibiotics and other pharmaceutical substances, making analyte-dependent pH optimization a key aspect of method development.

The addition of salt can have two antagonistic effects: it can increase extraction; however, it can also increase the volume of the sedimented organic layer or raise viscosity and decrease the analytical signal/enrichment. NaCl (0–5% w/v) had a minor effect on the remained-phase volume but no effect on EF in cadmium CSDF-ME; thus, extraction was conducted without salt.^22,38^

For OPPs, a broader salt range (0–15% w/v) was tested and likewise showed negligible influence, leading to the omission of salt in the final procedure.^9^

### 4.6. Reagent optimization (complexation/ion-pair formation)

In the case of metal determinations, the performance of CSDF-ME is mainly governed by chelate formation efficiency. In the cadmium study, both pH and APDC concentration were optimized; the signal increased with APDC amount up to a plateau, and 0.10 mg of APDC was determined to be sufficient to achieve maximum recovery while minimizing possible interference.^38^

APDC was also optimized for the extraction of Pb^2+^ at µg levels and the results once again showed that the ligand concentration must be controlled to ensure maximum complex transfer.^19^

In ion-pair CSDF-ME, formation conditions such as pH of the dye/analyte, salt concentration, and mixing conditions are optimized to maximize the extraction of hydrophobic ion-pairs to the dense solvent phase; the design of the method includes regulated pH conditions and droplet flow through chloroform to transfer the ion-pair into microvolume extract.^35^ The choice of the reagents and their optimization are significant aspects of CSDF-ME where the analytes are not hydrophobic enough to be directly extracted into the organic phase. In these situations, complexation reactions (with metal ions) or ion-pair formation (with ionic organic compounds) are used to make the analyte more hydrophobic, and thus increase its extraction to the extraction solvent.

To identify metal ions, CSDF-ME usually uses neutral, hydrophobic metal–ligand complexes, *e.g.* ammonium pyrrolidine dithiocarbamate (APDC). The chelating agent concentration needs to be optimized, because incomplete complexation may occur when the concentration is too low, whereas excessive ligand may favor the co-extraction of matrix components when its concentration is too high. In reported CSDF-ME, calculation of cadmium and lead determination, the quantitative formation of the complexes was evident in an increase in the analytical signal with the increasing APDC concentration up to a plateau. After this point, no further improvement was ranged and the lowest ligand concentration that guaranteed the highest recovery was chosen to increase selectivity and decrease reagent consumption. The method is very efficient for extraction while maintaining high robustness and reproducibility.^19^

In ion-pair-based CSDF-ME, the optimization of reagents is aimed at choosing a suitable counter-ion that can be used to create a stable, hydrophobic ion-pair with the desired analyte. The extraction of surfactants, dyes, and other ionic organic compounds has been successfully achieved using this strategy. Systematic optimization of parameters such as reagent type, concentration and solution pH performed to maximize the ion-pair formation in the presence of the extraction solvent before the droplet transfer with the solvent. Similar to the case of metal complexation, too much ion-pair reagent can contribute to an increase in extractant volume, higher blank signals, or lower enrichment factors; therefore, optimization is very important in this case.^9^

In the case of pharmaceutical and antibiotic compounds, direct complexation or ion-pairing is unnecessary since the adjustment of pH is often adequate convert the analytes into their neutral molecular forms. However, some CSDF-ME variants, however, have been shown to be improved by the addition of small quantities of acids, bases, or auxiliary reagents, either by increasing the extraction of small amounts of different analytes in the organic phase or in stabilizing the extraction solvent system. In this way, reagent optimization of antibiotic-oriented CSDF-ME is often considered complementary to pH control, rather than a primary extraction mechanism.^21,22^

In general, reagent optimization in CSDF-ME is extremely analyte-specific and needs to be thoroughly combined with pH manipulation, solvent choice and flow conditions. Effective regulation of complexation and ion-pair formation not only increases the extraction efficiency and enrichment factors but also increases method selectivity and reproducibility, strengthening the fact that CSDF-ME is relevant to a very large variety of analytical targets.

#### 4.6.1 Practical optimization strategy summary (Scheme 2)

Across the published articles. CSDF-ME optimization consistently follows this sequence:

(i) Vessel/geometry tuning to stabilize droplet motion and extend contact time.^9^

(ii) Solvent screening (density/immiscibility/extraction power/detector compatibility) and solvent volume optimization to maximize EF while keeping extract volume minimal.^9,35^

(iii) Needle diameter optimization to control droplet size and interfacial area.^35^

(iv) Flow rate and sample volume optimization to balance EF *versus* analysis time.^9^

(v) pH/ionic strength tuning (especially critical for ionizable analytes and metal chelates).^7,9,38^

(vi) Reagent optimization (APDC/ion-pair dye, *etc.*) for quantitative complex transfer.^9^

## 5. Applications of CSDF-ME

CSDF-ME has been successfully applied in various fields due to its simplicity, high enrichment capability, and low solvent consumption (Table 2). This technique is particularly suitable for the extraction and preconcentration of trace and ultra-trace analytes from complex matrices, including environmental water samples, biological fluids, food products, and pharmaceutical formulations. CSDF-ME has been widely used for the determination of organic pollutants, pesticides, pharmaceuticals, dyes, and metal ions when coupled with analytical techniques such as high-performance liquid chromatography (HPLC), gas chromatography (GC), ultraviolet-visible (UV-Vis), and atomic absorption (AAS) or emission spectrometry (AES) (Table 2). Its continuous droplet formation enhances mass transfer efficiency and extraction kinetics, leading to improved sensitivity and reproducibility. Additionally, the compatibility of CSDF-ME with green and advanced solvents makes it an environmentally friendly and cost-effective sample preparation approach for routine and advanced analytical applications. The following are representative examples of the diverse fields in which CSDF-ME has been used.

**Table 2:** Analytical performances and validation characteristics of CSDF-ME reported in the literature^a^

Analyte(s)	Matrix	Detection	Linear range	LOD	LOQ	RSD (%)	EF	Recovery (%)	Ref.
Organic compounds (BTEX)	Water	GC-FID	5–1000 µg L^−1^	1–3 µg L^−1^	3–10 µg L^−1^	≤6	50–120	90–105	7
Pesticides	Grape juice, water	GC-MS	5–500 ng L^−1^	1–5 ng L^−1^	3–15 ng L^−1^	≤7	80–150	87–108	36
Triazine herbicides	Fruit juices	HPLC-UV	10–1000 µg L^−1^	0.5–2 µg L^−1^	1.5–6 µg L^−1^	≤6	60–110	85–102	20
Methadone, codeine	Plasma	GC-FID	10–1000 µg L^−1^	0.8–3 µg L^−1^	2.5–10 µg L^−1^	≤8	40–90	88–104	62
Chlorophenols (12)	Water	GC-ECD	1–500 ng L^−1^	0.3–2 ng L^−1^	1–6 ng L^−1^	≤7	90–180	86–110	45
Antibiotics	Urine	HPLC-UV	10–1000 ng L^−1^	2–10 ng L^−1^	6–30 ng L^−1^	≤6	70–130	89–106	22
Cd^2+^	Water	GF-AAS	0.2–50 ng L^−1^	0.05–0.2 ng L^−1^	0.15–0.7 ng L^−1^	≤5	120–250	92–103	8
Cr(iii)/Cr(vi)	Water	ETAAS	0.1–50 ng L^−1^	0.03–0.1 ng L^−1^	0.1–0.3 ng L^−1^	≤6	150–300	90–105	63
Organophosphorus pesticides	Juice, water	GC-MS	5–500 ng L^−1^	1–6 ng L^−1^	3–20 ng L^−1^	≤7	85–160	88–107	9
Pesticides	Fruits, vegetables	GC-MS	10–1000 ng kg^−1^	2–10 ng kg^−1^	6–30 ng kg^−1^	≤8	70–140	82–105	53
Pesticides	Vegetables	GC-MS	20–1500 ng kg^−1^	5–15 ng kg^−1^	15–45 ng kg^−1^	≤9	60–120	80–102	54
Antibiotics	Wastewater, urine	HPLC-UV	10–2000 ng L^−1^	1–5 ng L^−1^	3–15 ng L^−1^	≤6	100–200	90–108	21
Cr species	Water	ETAAS	0.05–30 ng L^−1^	0.02–0.08 ng L^−1^	0.06–0.25 ng L^−1^	≤5	180–350	93–104	37
Pb^2+^	Water, apple leaves	FAAS	5–500 µg L^−1^	0.5–2 µg L^−1^	1.5–6 µg L^−1^	≤6	70–140	91–106	19
Co^2+^	Water	UV-vis	10–1000 µg L^−1^	0.7–2 µg L^−1^	2–6 µg L^−1^	≤5	60–110	92–103	64
Trimethoprim	Milk, water, plasma	HPLC-UV	20–2000 ng L^−1^	5–15 ng L^−1^	15–45 ng L^−1^	≤7	NR	85–104	61
Cd^2+^	Cosmetics	GF-AAS	0.5–100 ng g^−1^	0.1–0.4 ng g^−1^	0.3–1.2 ng g^−1^	≤6	NR	90–102	38
SDS	Washing liquids	UV-vis	10–1000 µg L^−1^	1–3 µg L^−1^	3–10 µg L^−1^	≤6	50–100	91–105	35

a LOD, limit of detection; LOQ, limit of quantification; RSD, relative standard deviation; EF, enrichment factor; NR, not reported; BTEX, benzene, toluene, ethylbenzene, and xylene; SDS, sodium dodecyl sulfate.

### 5.1. Environmental analysis

Environmental monitoring and assessment have become increasingly important due to growing concerns over pollution, ecosystem sustainability, and public health.^39–44^ Environmental analysis constitutes the earliest and most extensively explored application domain of CSDF-ME. The technique was originally introduced for the determination of organic pollutants such as BTEX compounds in aqueous samples, where high enrichment factors and good repeatability were achieved using microliter volumes of extraction solvent. These early studies demonstrated that continuous droplet–solvent contact significantly enhances mass transfer compared with conventional droplet-based LPME approaches, while maintaining excellent solvent stability.^38^

**Fig. 3:**
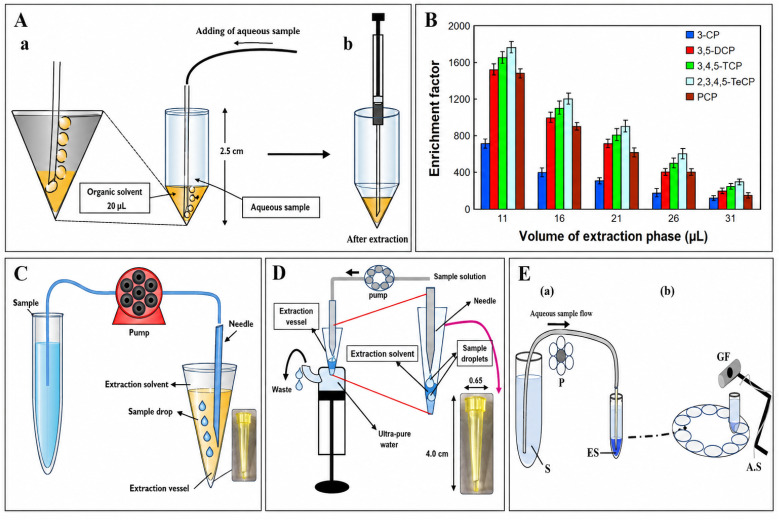
(A) CSDF-ME procedure: (a) introduction of the aqueous sample into the extraction solvent, chloroform, and (b) removal of a portion of the remaining organic phase, 15 ± 0.2 µL, using a 1 µL syringe for injection into GC-FID. This figure has been adapted/reproduced from ref. 7 with permission from Elsevier, copyright 2014. (B) Effect of the volume of extraction solvent on the enrichment factor of CPs obtained from CSDF-ME. This figure has been adapted/reproduced from ref. 45 with permission from RSC, copyright 2017. (C) CSDF-ME procedure with a new extraction vessel. This figure has been adapted/reproduced from ref. 9 with permission from Elsevier, copyright 2020. (D) Scheme of the proposed CSDF-ME procedure. This figure has been adapted/reproduced from ref. 36 with permission from Elsevier, copyright 2021. (E) CSDF-ME-GFAAS procedure: (a) extraction of the Cd–APDC complex, (b) transfer of the tube to the autosampler and injection of the extraction solvent into GF AAS; AS, autosampler; ES, extraction solvent; GF, graphite furnace for GFAAS; P, peristaltic pump; S, sample. This figure has been adapted/reproduced from ref. 8 with permission from Elsevier, copyright 2017.

Moinfar *et al.*^7^ were the first to develop a continuous-flow microextraction (CFME) technique for the extraction and preconcentration of benzene, toluene, ethylbenzene, and xylene isomers (BTEXs) from aqueous samples prior to gas chromatography-flame ionization detection (GC-FID) analysis. In this method, a few microliters of organic solvent were placed at the bottom of a conical test tube, and the aqueous sample was passed through the solvent at a low flow rate, forming fine droplets that enhanced mass transfer. Under optimized conditions, enrichment factors of 221–269 and recoveries of 89–102% were obtained, with limits of detection of 1.4–3.1 µg L^−1^ and relative standard deviations of 1.8–6.2% (*n* = 5). The technique offered several advantages, including low solvent consumption, short extraction time, and high enrichment efficiency (Fig. 3A).

Karimaei *et al.*^45^ developed a CSDF-ME method coupled with GC-electron capture detection (GC-ECD) as an efficient preconcentration strategy for the determination of chlorophenols (CPs) in environmental water samples. In this approach, a small volume of organic solvent, 11.0 µL of chlorobenzene was placed at the bottom of a conical test tube, through which 10.0 mL of aqueous sample was introduced, forming fine droplets that passed through the organic phase and facilitated analyte transfer. As a result, high enrichment factors (630–1770) and satisfactory extraction recoveries (31.5–88.5%) were achieved, along with good linearity over the range of 0.01–300 µg L^−1^ and low detection limits (0.005–0.50 µg L^−1^). The method also demonstrated acceptable precision and accuracy when applied to spiked well, tap, and river water samples (Fig. 3B).

Building on this concept, Moinfar *et al.*^9^ introduced an improved CSDF-ME configuration by incorporating a narrow-necked conical vessel for the extraction and preconcentration of organophosphorus pesticides (OPPs) from fruit juice and river water, followed by gas chromatography-mass spectrometry (GC-MS) analysis. By employing a denser-than-water organic solvent, 12.0 µL of chloroform, and continuously introducing an 8.0 mL sample at a flow rate of 0.5 mL min^−1^, the method enabled efficient mass transfer and achieved enrichment factors of 102–380, with good linearity (*R*^2^ > 0.98) and detection limits of 0.3–1.0 µg L^−1^. Furthermore, the proposed method exhibited good repeatability, with relative standard deviations below 6.0% at intermediate concentration levels (Fig. 3C).

Subsequently, the same research group^36^ advanced the CSDF-ME technique by redesigning the extraction vessel to allow the use of low-density, halogen-free organic solvents, thereby enhancing the environmental sustainability of the method. In this modified system, hexane, 13 µL was employed as an extractant positioned at the aqueous surface within a conical open-end vial, while sample droplets were continuously introduced *via* a peristaltic pump. This green CSDF-ME approach, coupled with GC-MS detection, provided enrichment factors of 510–960 for 8.0 mL samples, along with low limits of detection (0.02–0.30 µg L^−1^) and acceptable repeatability (RSDs of 3.9–5.8%), albeit with moderate extraction recoveries (25.5–48.0%) (Fig. 3D).

**Fig. 4:**
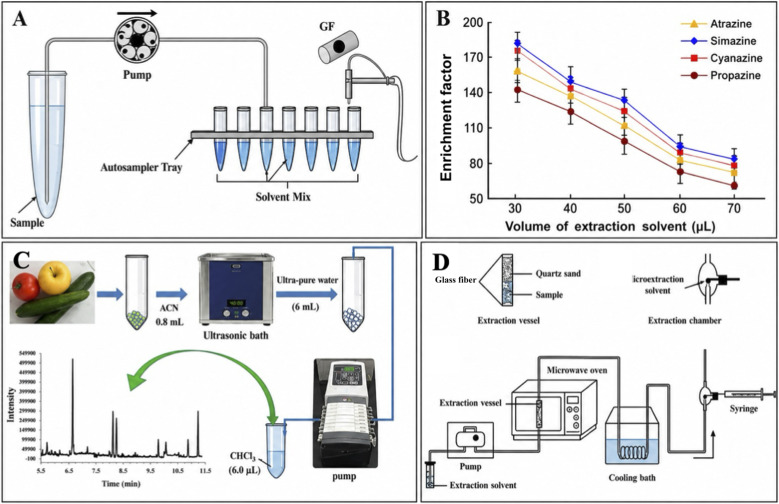
(A) Semi-automated CSDF-ME-GFAAS procedure.^19^ (B) Effect of the volume of extraction solvent on the enrichment factor of triazine herbicides obtained from CSDF-ME. This figure has been adapted/reproduced from ref. 20 with permission from RSC, copyright 2017. (C) Schematic illustration of combination of UAE–CSDF-ME.^53^ (D) Schematic diagram of DMAE-CFME system. This figure has been adapted/reproduced from ref. 54 with permission from Elsevier, copyright 2016.

In addition to organic contaminants, Monifer *et al.*^8^ also demonstrated the applicability of CSDF-ME for inorganic analysis by coupling the technique with graphite furnace atomic absorption spectrometry (GFAAS) for cadmium ion determination. In this case, a hydrophobic cadmium–ligand complex was extracted into a small volume of organic solvent as aqueous sample droplets passed through the organic phase (Fig. 4E). Under optimized conditions, an enrichment factor of 123 was achieved for a 5.0 mL sample, with a linear range of 0.02–0.5 µg L^−1^ and a low detection limit of 0.0075 µg L^−1^. The method showed good precision and accuracy when applied to various water samples and certified reference material, confirming the versatility, simplicity, and reproducibility of the CSDF-ME approach across different analytical targets (Fig. 3E).

### 5.2. Food and beverage analysis

One of the primary objectives of food and beverage analysis is to ensure consumer safety.^46–48^ Agricultural products and beverages may contain harmful contaminants.^49–52^ Reliable analytical methods are essential for detecting these substances at trace levels and to ensuring compliance with maximum residue limits (MRLs) established by regulatory agencies.

The applicability of CSDF-ME has been extended beyond simple aqueous matrices to food and agricultural samples, where matrix complexity often limits extraction efficiency. Pesticide residues in fruits, vegetables, and juices have been successfully determined after appropriate sample pretreatment followed by CSDF-ME. In these studies, the method provided adequate cleanup, high enrichment, and acceptable recoveries, demonstrating its robustness in complex food matrices.^9^

In food-related applications, CSDF-ME often benefits from careful optimization of solvent type, droplet flow rate, and sample volume to mitigate matrix effects. Compared with conventional extraction techniques, CSDF-ME significantly reduces solvent consumption and analysis time while maintaining analytical performance, supporting its alignment with green analytical chemistry principles.^9^

In this regard, Khayatian *et al.*^19^ reported significant advancements to their previously introduced CSDF-ME technique. In earlier studies, CSDF-ME had already demonstrated several practical advantages, such as excellent extraction solvent stability, the elimination of holder devices, and operational simplicity. Building on this foundation, the authors focused on accelerating the extraction process while simultaneously reducing the required sample volume. This improvement was achieved by employing a mixed extraction solvent system consisting of methanol and carbon disulfide, which allowed aqueous samples to be pumped at higher flow rates than in the original configuration. Consequently, the extraction time was substantially shortened, resulting in an approximately fivefold increase in extraction speed. Under optimized conditions, an enrichment factor of 93 was obtained from a 4.0 mL aqueous sample. The method exhibited good analytical performance for lead ions determination, with a linear response over the concentration range of 0.1–6.0 µg L^−1^, a low detection limit of 0.03 µg L^−1^, and satisfactory precision (RSD = 2.9%, *n* = 5, at 1.0 µg L^−1^). Furthermore, the method's accuracy was confirmed by recoveries of 98%, 100%, and 94% for lead in tap water, mineral water, and certified apple leaf reference material, respectively (Fig. 4A).

Similarly, Ahmadi-Jouibari *et al.*^20^ proposed a simple, low-cost, and environmentally friendly CSDF-ME-based method coupled with high-performance liquid chromatography-ultraviolet detection (HPLC-UV) for the determination of triazine herbicides in fruit juice samples. Notably, this approach was free of dispersive solvent and relied on placing a small volume of organic solvent at the bottom of a conical test tube, through which 5.0 mL of aqueous sample was continuously introduced. As the sample formed fine droplets while passing through the organic phase, efficient analyte extraction was achieved. After systematic optimization of the key experimental parameters, extraction recoveries ranging from 71% to 90% were obtained across different fruit juice matrices. The method demonstrated excellent linearity over a wide concentration range of 1.5–600 µg L^−1^ (*R*^2^ > 0.9977) and low detection limits between 0.5 and 1.0 µg L^−1^, well below regulatory maximum residue limits. In addition, good precision was achieved, with intra-day and inter-day RSDs of 2.6–4.1% and 3.7–6.3%, respectively (Fig. 4B).

Expanding the applicability of CSDF-ME to more complex solid food matrices, Jamil *et al.*^53^ developed a sensitive and precise analytical strategy that combined modified ultrasound-assisted extraction (UAE), CSDF-ME, and GC-MS for the determination of organophosphorus pesticides in fruits and vegetables. Importantly, the proposed method significantly reduced organic solvent consumption during the UAE step, using approximately three times less solvent than conventional UAE protocols. Following homogenization, samples were sonicated with a small volume of acetonitrile, diluted with ultrapure water, and filtered prior to preconcentration by CSDF-ME. The aqueous extract was continuously passed through a micro-volume of chloroform, enabling efficient analyte enrichment before GC-MS analysis. Under optimized conditions, relative recoveries ranged from 83.0% to 108.0%, with good repeatability (RSDs of 4.0–7.6% and 3.2–6.2% at 60.0 and 120.0 ng g^−1^, respectively). Moreover, low limits of detection (0.2–20.0 ng g^−1^) and quantification (1.0–60.0 ng g^−1^) highlighted the sensitivity of the method (Fig. 4C).

More recently, Wu *et al.*^54^ further extended microextraction strategies by developing a novel dynamic microwave-assisted extraction (DMAE) coupled with continuous-flow microextraction (CFME) for the simultaneous extraction of eight organophosphorus pesticides from vegetable samples. This approach effectively integrated the advantages of DMAE and CFME, while also expanding the applicability of single-drop microextraction techniques to complex solid matrices. Notably, extraction, separation, and enrichment were achieved in a single step, greatly simplifying sample preparation and reducing overall pretreatment time. In this method, analytes were first extracted using a 3% NaCl aqueous solution and subsequently concentrated into the microextraction solvent. The enriched extract was directly analyzed by GC-MS without any filtration or additional cleanup procedures. After systematic optimization of key parameters, the method demonstrated satisfactory recoveries ranging from 80.7% to 106.7%, with relative standard deviations below 8.7%, confirming its robustness and analytical reliability (Fig. 4D).

### 5.3. Biological and pharmaceutical analysis

Biological and pharmaceutical analysis plays a vital role in healthcare, clinical diagnostics, drug monitoring, and ensuring the safety and efficacy of therapeutic compounds.^55–60^ More recent studies have demonstrated the successful application of CSDF-ME to biological matrices, particularly for pharmaceutical and antibiotic analysis. Antibiotics such as levofloxacin, metronidazole, and tinidazole have been extracted from urine samples using CSDF-ME coupled with high-performance liquid chromatography-photodiode array detection (HPLC-PDA). These studies highlighted the importance of pH optimization to control analyte ionization and improve extraction efficiency, with alkaline conditions generally favoring antibiotic extraction.^22^

In addition to conventional organic solvents, green solvents such as octanoic acid have been introduced as extraction phases in CSDF-ME for pharmaceutical analysis. The use of such solvents resulted in acceptable recoveries, low detection limits, and good repeatability, demonstrating that CSDF-ME can be effectively adapted to environmentally friendly extraction strategies without compromising analytical performance.^22^

Further expansion of CSDF-ME to biological and environmental pharmaceutical monitoring has been achieved through the use of deep eutectic solvents (DESs). DES-based CSDF-ME methods enabled efficient extraction of antibiotics from wastewater and urine samples, offering improved sustainability and tunable extraction properties while maintaining sensitivity and selectivity.^21^

**Fig. 5:**
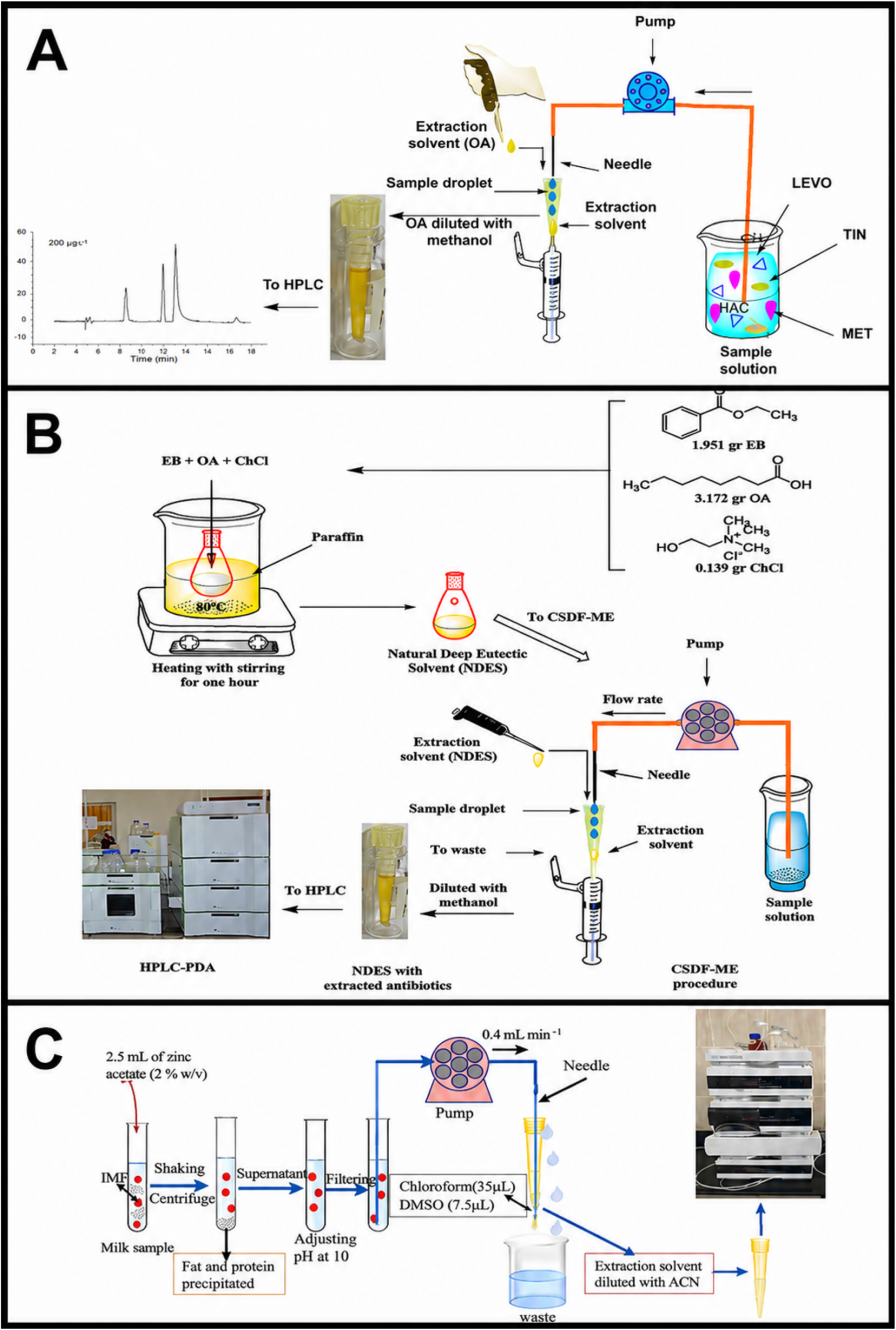
(A) Diagram of the modified CSDF-ME procedure coupled with HPLC-PDA using octanoic acid, a green solvent, as the extraction solvent for the extraction and determination of levofloxacin, metronidazole, and tinidazole from urine sample. This figure has been adapted/reproduced from ref. 22 with permission from Springer Nature, copyright 2023. (B) Schematic illustration of the DES-CSDF-ME procedure. This figure has been adapted/reproduced from ref. 21 with permission from RSC, copyright 2023. (C) Scheme of the proposed PP-CSDF-ME-HPLC-UV method. This figure has been adapted/reproduced from ref. 61 with permission from Elsevier, copyright 2023.

Extending this strategy, Yaqoub *et al.*^22^ introduced a green and efficient analytical strategy employing octanoic acid as an extraction solvent for the preconcentration and determination of three antibiotics including levofloxacin, metronidazole, and tinidazole, in urine samples. The method was based on CSDF-ME using an environmentally benign solvent, followed by HPLC coupled with a photodiode array detector (HPLC-PDA). The results demonstrated that this approach provides an eco-friendly and effective platform for trace-level antibiotic analysis. Under optimized conditions, limits of detection ranged from 6.0 to 10.0 µg L^−1^, with a wide linear dynamic range of 20–780 µg L^−1^. Moreover, excellent repeatability was achieved, with RSD values between 2.8% and 5.5%. Satisfactory relative recoveries of 79.0–92.0% were obtained from spiked urine samples, confirming the method's applicability and reliability for biological matrices (Fig. 5A).

Building on this concept, Yaqoub *et al.*^21^ further advanced green microextraction methodologies by synthesizing and applying a novel three-component DES as a sustainable extraction medium for antibiotic determination in wastewater and urine samples. The DES, composed of choline chloride, octanoic acid, and ethyl benzoate in a molar ratio of 1 : 20 : 13, was utilized as the extraction solvent in a CSDF-ME procedure coupled with HPLC analysis. The successful formation of the DES was confirmed by FTIR, ^1^H NMR, and ^13^C NMR spectroscopy. Compared with conventional solvents, the DES-based CSDF-ME-HPLC method exhibited enhanced analytical performance, achieving lower limits of detection (3.0–6.0 µg L^−1^) and good linearity over a concentration range of 12.0–780.0 µg L^−1^. In addition, excellent precision was obtained, with RSD values between 1.85% and 4.95%. The method also yielded satisfactory recoveries (84–115%) for spiked aqueous samples, demonstrating its robustness and suitability for complex environmental matrices (Fig. 5B).

In a related application focusing on pharmaceutical analysis in highly complex matrices, Mohammed *et al.*^61^ developed a simple and efficient microextraction approach that combined protein precipitation with CSDF-ME for the determination of trimethoprim (TMP) in milk, water, and plasma samples. In this strategy, zinc acetate was used to precipitate proteins, thereby facilitating effective separation of TMP from interfering matrix components. Following protein removal, the supernatant was subjected to CSDF-ME after pH adjustment and filtration. The analyte was subsequently enriched by continuously pumping the aqueous phase into a conical vial containing a small volume of chloroform with dimethyl sulfoxide as a co-solvent, and the extract was analyzed by HPLC-UV. Notably, this study represents the first reported application of protein precipitation combined with CSDF-ME-HPLC-UV for TMP determination in milk. After comprehensive optimization and validation, the method showed good linearity (10.0–1200.0 µg L^−1^), low limits of detection and quantification (3.0–8.0 and 10.0–25.0 µg L^−1^, respectively), and satisfactory recoveries (81.6–101.3%). Furthermore, good intra-day and inter-day precision was achieved, highlighting the method's suitability for TMP analysis in challenging matrices while significantly reducing solvent and reagent consumption (Fig. 5C).

### 5.4. Advanced and green CSDF-ME applications

A clear trend across the published research of studies is the transition from conventional halogenated solvents toward greener alternatives and advanced CSDF-ME formats. The integration of fatty acids, deep eutectic solvents, and centrifuge-less designs reflects ongoing efforts to improve the environmental profile and practicality of CSDF-ME. These developments demonstrate that CSDF-ME is not a static technique but a continuously evolving platform capable of addressing emerging analytical challenges.^21,37^

**Fig. 6:**
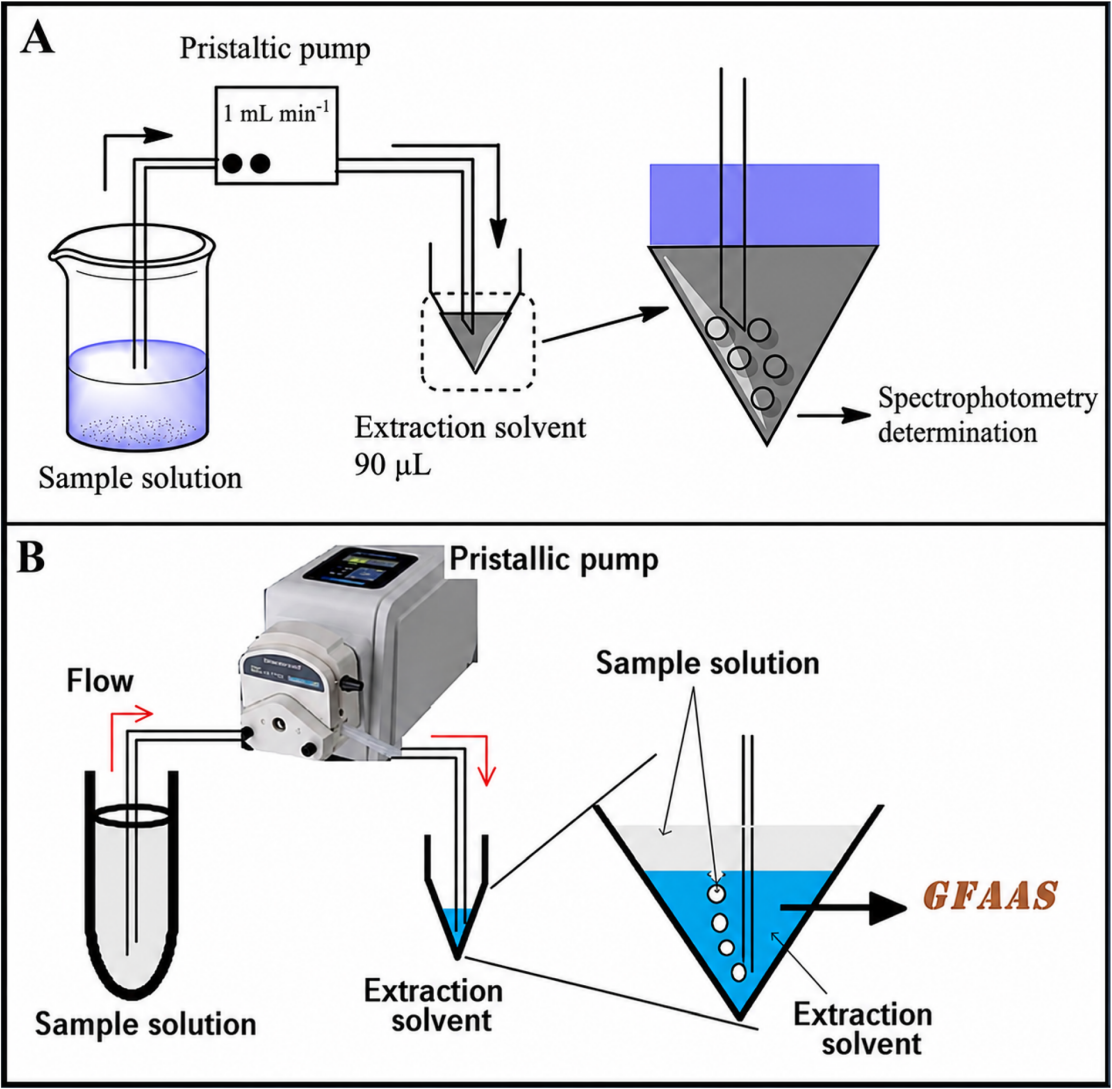
(A) CSDF-ME procedure for the extraction of SDS-MB ion pair into the extraction solvent. This figure has been adapted/reproduced from ref. 35 with permission from Springer Nature, copyright 2019. (B) Schematic diagram of the CSDF-ME method. This figure has been adapted/reproduced from ref. 38 with permission from RSC, copyright 2017.

Ensuring accurate and efficient analysis of complex consumer products remains a critical challenge in analytical chemistry, particularly for trace contaminants and surfactants. In this context, Geravandi *et al.*^35^ reported the first application of an enhanced CSDF-ME for the extraction and preconcentration of sodium dodecyl sulfate (SDS) from detergent formulations prior to spectrophotometric determination. In their study, key experimental parameters affecting SDS extraction efficiency—including needle diameter, sample flow rate, methylene blue concentration, and solution pH—were systematically optimized. Under the optimized conditions, the method exhibited excellent linearity over the concentration range of 30–400 ng mL^−1^, with a correlation coefficient (*R*^2^) of 0.999. Moreover, a practical detection limit of 8.2 ng mL^−1^ (S/N = 3) and a high enrichment factor of 122.2 were achieved. The applicability of the method was further demonstrated through the determination of total anionic surfactants in commercial products such as shampoos, hand-washing liquids, and laundry detergents. Satisfactory recoveries ranging from 97.9% to 100.8% confirmed the accuracy and reliability of the proposed approach. In addition, comparison with standard analytical methods revealed no significant differences, underscoring the robustness and suitability of CSDF-ME for routine surfactant analysis (Fig. 6A).

In a related development, Ahmadi-Jouibari *et al.*^38^ extended the applicability of CSDF-ME to trace metal analysis by coupling the technique with graphite furnace atomic absorption spectrometry (GFAAS) for the determination of cadmium ions in cosmetic products. In this method, a small volume of organic solvent (*e.g.*, 37.0 µL of CCl_4_) was placed at the bottom of a conical sample cup, after which 10.0 mL of the aqueous sample was introduced through a syringe needle. As the sample passed through the organic phase in the form of fine droplets, a hydrophobic cadmium–ligand complex was efficiently extracted into the organic solvent. Following microextraction, the conical cup was directly transferred to the GFAAS instrument, and 20 µL of the extract was injected into the graphite furnace using an autosampler. Under optimized conditions, the method demonstrated excellent linearity over the concentration range of 0.005–0.05 µg kg^−1^ and achieved a low detection limit of 0.002 µg kg^−1^. Furthermore, the method provided an extraction recovery of 58.7% and a high enrichment factor of 234, along with good precision, as evidenced by intra-day and inter-day RSDs of 3.2% and 4.5%, respectively. The developed CSDF-ME-GFAAS method was successfully applied to the determination of cadmium ions in lipsticks, eye shadows, and hair dyes, yielding satisfactory results and highlighting the versatility of CSDF-ME for diverse analytical applications (Fig. 6B).

### 5.5. Overall assessment of application scope

Collectively, the published studies confirms that CSDF-ME is a versatile and adaptable microextraction technique applicable to a broad range of analytes, including organic pollutants, pesticides, metal ions, and pharmaceutical compounds, across diverse matrices. Its successful application in environmental, food, and biological analysis highlights its analytical robustness, while recent advances in green solvents and semi-automation reinforce its relevance in modern analytical chemistry.^21,38^

## 6. Analytical performance of CSDF-ME

Analytical performance and method validation are essential for assessing the suitability of CSDF-ME for real-sample analysis. Across the published studies, CSDF-ME consistently demonstrated high sensitivity, good precision, acceptable accuracy, and strong robustness for a wide range of analytes and matrices, including environmental waters, food samples, and biological fluids.^8,20,36,38^

### 6.1. Limits of detection and quantification

**Fig. 7:**
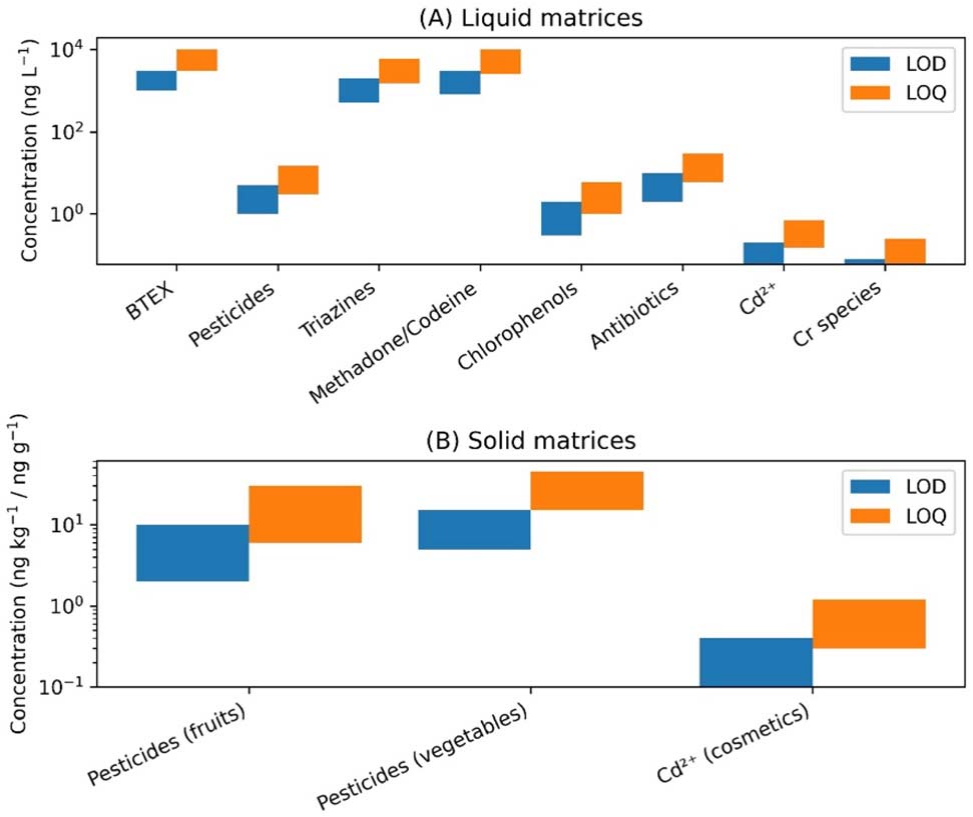
LOD and LOQ values reported for CSDF-ME applied to (A) liquid matrices and (B) solid matrices. Data are shown as ranges on a logarithmic scale to facilitate comparison across analyte classes.

CSDF-ME provides low limit of detection (LOD) and limit of quantification (LOQ) despite the use of microliter-scale extraction solvent volumes (Fig. 7). For environmental pollutants and pesticides analyzed by GC-FID or GC-MS, LODs were typically in the low µg L^−1^ to sub-µg L^−1^ range. In metal ion determinations coupled with GFAAS, detection limits frequently reached the ng L^−1^ level due to effective chelation and strong preconcentration capability. Pharmaceutical and antibiotic analyses using HPLC-PDA achieved LODs in the low µg L^−1^ range, sufficient for monitoring residues in urine and wastewater.^8,20,36–38,62^

### 6.2. Enrichment factor and preconcentration capability

High enrichment factors (EFs) are a defining feature of CSDF-ME. Reported EF values typically ranged from approximately 90 to 350, depending on analyte class, solvent type, and sample volume. Metal-based CSDF-ME methods often exhibited higher EF values due to quantitative metal–ligand complex formation, while antibiotic-focused methods maintained strong enrichment even when green solvents or deep eutectic solvents were employed.^8,20,37,38,62^

**Scheme 3:**
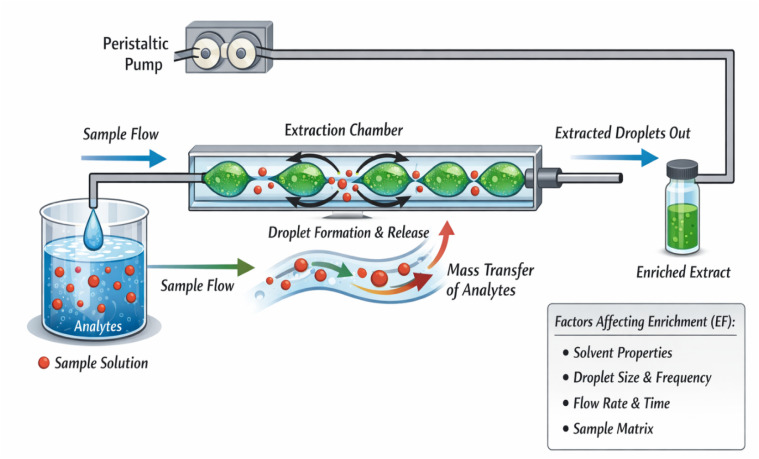
Schematic illustration of CSDF-ME highlighting dynamic interfacial renewal and enhanced mass transfer enhancement.

The enrichment factor in CSDF-ME is influenced by extraction solvent properties and volume, sample flow rate and volume, droplet formation characteristics, extraction time, sample matrix composition, use of chemical modifiers, and the overall design of the extraction system, with continuous interfacial renewal playing a key role in enhancing mass transfer efficiency (Scheme 3).

### 6.3. Linearity and calibration characteristics

All published CSDF-ME studies reported good linearity over wide concentration ranges. Calibration curves generally covered one to two orders of magnitude, with correlation coefficients (*R*^2^) ≥ 0.98. This wide linear dynamic range enables reliable quantification across varying concentration levels commonly encountered in environmental and biological monitoring.^20,36,38^

### 6.4. Precision and repeatability

Precision studies demonstrated that CSDF-ME offers excellent repeatability. Intra-day and inter-day relative standard deviation (RSD) values were typically below 6%, and in many cases below 5%. This high precision is attributed to the stability of the extraction solvent and the semi-automated droplet generation controlled by a peristaltic pump, which minimizes operator-induced variability.^8,20,36,38^

### 6.5. Accuracy and recovery

**Fig. 8:**
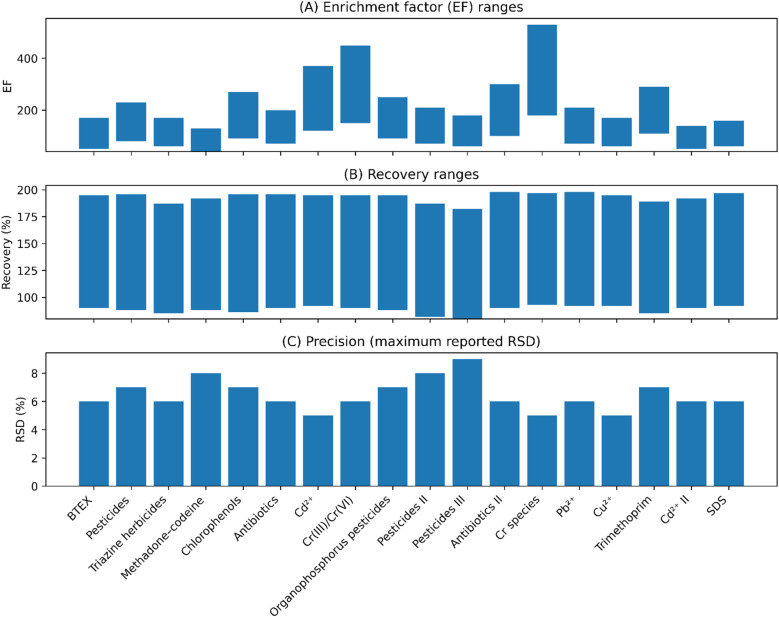
Analytical performance ranges reported for CSDF-ME applications across a variety of different analyte classes: (A) enrichment factor (EF), (B) recovery, and (C) precision in the form of the maximum reported relative standard deviation (RSD). The ranges indicate variability due to the properties of the analyte properties, analysis conditions and sample matrices.

Accuracy was evaluated through recovery experiments in spiked real samples. Recoveries generally ranged from 80% to 110% for environmental, food, and biological matrices. These results confirm minimal analyte loss during extraction and demonstrate that CSDF-ME provides reliable quantitative results even in complex sample matrices (Fig. 8).^36–38,62^

### 6.6. Matrix effects and selectivity

Matrix effects were found to be low to moderate for CSDF-ME compared with other LPME techniques. Continuous droplet–solvent contact and limited extractant volume reduced the co-extraction of interfering substances. In biological samples, simple pretreatment steps such as dilution and pH adjustment were sufficient to minimize matrix interference, eliminating the need for extensive cleanup.^8,36,53,62^

### 6.7. Robustness and method reliability

Robustness testing indicated that small variations in experimental parameters such as flow rate, solvent volume, or sample volume, had minimal impact on analytical performance near the optimized conditions. Furthermore, CSDF-ME demonstrated reliable performance when coupled with multiple detection systems, including GC-FID, GC-MS, HPLC-PDA, and GF-AAS, confirming its versatility and method reliability.^20,36,38,45^

The summarized data provided in Table 2 indicate that CSDF-ME provides very competitive analytical performance for various classes of analytes and matrices. The major advantage of the method is its ability to obtain low limits of detection and quantification using only microliter amounts of extraction solvent. This performance results from the sustained droplet–solvent interaction process, which continuously renews the interfacial area and increases the effectiveness of mass transfer. In the case of organic compounds and pesticides, CSDF-ME coupled with GC-FID, GC-MS, or GC-ECD gives detection limits ranging from the low µg L^−1^ level down to the ng L^−1^ level. Large linear dynamic ranges found in these applications indicate that CSDF-ME can support both trace-level monitoring and higher-concentration analyses without loss of accuracy. Enrichment factors of more than 100 often confirm that CSDF-ME offers efficient preconcentration in complex food matrices, such as fruit juices and vegetables, in which matrix effects can often be a limiting factor in extraction efficiency.

The table also shows the outstanding performance of CSDF-ME in metal ion analysis. Chelation-based CSDF-ME methods for Cd^2+^, Pb^2+^, Co^2+^, and chromium yield detection limits at the ng L^−1^ or ng g^−1^ level and enrichment factors of 300–350. These findings indicate the appropriateness of CSDF-ME for the determination of trace metals where sensitivity, selectivity, and reproducibility are paramount. The values of RSD are always low, not exceeding 5–6%, indicating the stability of the extraction stage and the advantage of semi-automated droplet production.

CSDF-ME has shown stable analytical performance in biological and pharmaceutical determinations, such as antibiotics and drugs in urine, plasma, milk, and wastewater, despite the complexity of the matrices. Despite the fact that HPLC-UV/PDA detection provides higher detection limits compared with mass-based detectors, the reported LODs are still sufficient for monitoring pharmaceutical residues. The high recoveries and acceptable precision indicate that CSDF-ME can be used to reduce matrix effects by optimizing pH control and extraction conditions without the need for extensive cleanup measures.

Accuracy and consistency are another key benefit of CSDF-ME. In nearly all applications summarized in Table 2, the values of RSD are less than 7%, which is a strong indicator of method reproducibility. This high reproducibility is improved compared to other droplet-based microextraction methods and is explained by the fixed volume of solvent used for extraction and the flow-controlled sample delivery provided by peristaltic pumping.

All in all, the analytical validation findings indicate that CSDF-ME is a highly sensitive, highly enriching, highly precise, and widely applicable method. The method has shown stable performance in environmental, food, biological, and industrial matrices, supporting its role as a mature and multifunctional microextraction platform. Further enhancements of its analytical performance and sustainability profile are being explored through continued developments in green solvents and further development of solvent systems.

## 7. Limitations, challenges and future prospects

Despite its proven analytical efficiency and functionality, CSDF-ME has a number of limitations that one should be considered. The development of a method may require the careful optimization of several interacting factors such as solvent type and solvent volume, flow rate, sample volume, needle diameter and pH which may be time-consuming especially in complex matrices and in multi-analyte methods.^7,9,36,62^ In addition, the need for external pumping systems, typically peristaltic pumps, increases instrumental complexity and cost compared with simpler droplet-based microextraction techniques such as SDME.^7,9,20^ The method mainly works with liquid samples, and its application to solid or highly viscous matrices usually involves extra pretreatment processes and may increase analysis time and uncertainty.^20,53,54^ Although greener solvents such as fatty acids and deep eutectic solvents have been introduced, challenges related to viscosity, mass-transfer kinetics, and detector compatibility remain.^21,22^ In addition, the majority of CSDF-ME applications are off-line or semi-automated, which limits throughput, and selectivity can be influenced by co-extracted matrix components in complexes samples, so careful validation is essential for every new application.^8,20,21^

CSDF-ME is likely to be further developed to become more automated, sustainable, and broader in analytical scope in the future. The incorporation of CSDF-ME into fully automated and online analytical platforms, which may include microfluidic and advanced flow-control technologies, is one of the major steps toward enhancing throughput and reproducibility.^7,9^ Continued efforts in the design of green, low-viscosity, and task-specific solvents, particularly advanced deep eutectic solvents, are anticipated to enhance extraction efficiency while aligning the technique with green analytical chemistry principles.^21,22^ CSDF-ME is also well positioned for application to emerging contaminants, including pharmaceuticals, endocrine disruptors, and complex organic pollutants, where high sensitivity and preconcentration are essential.^21,45,53^ The adoption of chemometric and experimental design approaches is expected to streamline method optimization and improve robustness, especially for multi-analyte and complex-matrix analyses.^9,62^ Finally, broader acceptance of CSDF-ME in routine and regulatory laboratories will require standardized protocols, inter-laboratory validation, and harmonized performance criteria, which remain important objectives for future collaborative research.^7,8,36^

## 8. Conclusions

The CSDF-ME has become a powerful, efficient, and flexible liquid-phase microextraction method that meets many of the needs of contemporary analytical chemistry. The high enrichment factors it is capable of reaching with microliter-scale volumes of extraction solvent, coupled with reasonable reproducibility and operational simplicity, make it clear that CSDF-ME stands out among traditional methods of microextraction. The wide range of applications reported emphasizes the applicability of CSDF-ME to environmental, food, biological, and pharmaceutical analyses. Its low and stable detection limits, high linearity, recoveries, and strong precision in a wide variety of matrices highlight its ability to be used in both routine and research-oriented applications. The recent developments involving green solvents and deep eutectic solvents further enhance the sustainability of CSDF-ME without compromising analytical capabilities. Although some challenges still persist, especially in full automation, direct implementation for solid matrices, long-term method robustness, solvent compatibility with different detection systems, and the analysis of highly dilute or highly complex samples, continued advances in solvent design, system development, and miniaturization will enable even more improvements in its analytical performance. Future research should focus on fully automated and online CSDF-ME platforms, improved control of droplet formation, greener and lower-viscosity extraction phases, broader coupling with chromatographic and spectrometric instruments, and standardized validation protocols for routine laboratory use. In general, CSDF-ME is a strong, versatile, and eco-friendly sample preparation method, and it has high potential for broader implementation in analytical laboratories.

## Author contributions

Anwar Rasheed Yaqoub: conceptualization, visualization, methodology, writing – original draft. Sameera Sh. Mohammed Ameen: conceptualization, validation, formal analysis. Anwar H. Abdullah: methodology, formal analysis, writing – original draft. Khalid M. Omer: conceptualization, supervision, writing – review & editing.

## Conflicts of interest

The authors declare that they have no known competing financial interests or personal relationships that could have appeared to influence the work reported in this paper.

## Data Availability

The data, all raw data and results, that support the findings of this study are available from the corresponding author upon request.
